# Factors associated with COVID-19 vaccine intent among Latino SNAP participants in Southern California

**DOI:** 10.1186/s12889-022-13027-w

**Published:** 2022-04-05

**Authors:** Vanessa P. Scott, Sarah Hiller-Venegas, Kate Edra, Joe Prickitt, Yesenia Esquivel, Blanca Melendrez, Kyung E. Rhee

**Affiliations:** 1 Department of Pediatrics, Division of Academic General Pediatrics, UC San Diego School of Medicine, San Diego, USA; 2 UC San Diego Center for Community Health, San Diego, USA; 3 Department of Pediatrics, Division of Child and Community Health, UC San Diego School of Medicine, 9500 Gilman Drive, MC 0874, La Jolla, CA 92093 USA

## Abstract

**Background:**

COVID-19 is significantly impacting the health and well-being of the country, particularly for ethnic minority populations and low-income groups. Our goal was to determine COVID-19 vaccination intent in a low-income, Latino population receiving aid from the Supplemental Nutrition Assistance Program (SNAP) in Southern California, and identify contributing factors and concerns.

**Methods:**

A cross-sectional, mixed-methods survey was conducted among participants in the Southern California Nutrition Incentives Program *(¡Más Fresco! More Fresh*). Only Latino respondents were included in this analysis. Primary outcome was vaccine intent trichotomized into: “definitely/likely yes”, “not sure/don’t know”, and “definitely/likely not.”

**Results:**

The majority of participants (*n* = 486) were female (93%), Spanish speaking (74%), with a median age of 40 years (IQR = 13). Approximately half (48%) reported they would get a COVID-19 vaccine, 39% were unsure, and 13% reported “definitely/likely not”. In the multivariable multinomial logistic regression model, participants with a household member with a COVID-19 health risk factor were more likely to be unsure about getting the vaccine. Participants who were primarily English speaking, did not receive the influenza vaccine last season, and reported not reading or talking about COVID-19 were more likely to report not intending to receive the vaccine. Many respondents were concerned about “side effects and ingredients”, and did not trust the vaccine development process, particularly with how fast it happened.

**Conclusion:**

Low-income Latinos in Southern California were generally hesitant to get a COVID-19 vaccine. Culturally sensitive vaccine promotion campaigns need to address the concerns of minority populations who experience increased morbidity and mortality from COVID-19.

**Supplementary Information:**

The online version contains supplementary material available at 10.1186/s12889-022-13027-w.

## Background

Coronavirus disease 2019 (COVID-19) has created world-wide challenges to healthcare systems and economies [1–3]. As of January 2022, COVID-19 has caused over 60 million cases and 835,000 deaths in the United States (US) [4]. Repercussions of the pandemic including overburdened hospitals and increased unemployment have highlighted widespread health disparities and the need to prevent COVID-19 with safe and effective vaccines.

Experts postulate 60–80% of the population need to be vaccinated to achieve herd immunity [5, 6]. However, increasing vaccine hesitancy (i.e., the delay in acceptance or refusal of vaccination despite availability of vaccination services [7]) and growing anti-vaccine sentiment may pose significant challenges [8–11]. Understanding why some groups are vaccine hesitant is imperative to develop effective public health messaging surrounding the new COVID-19 vaccines [12, 13].

Historically, Latino populations have lower vaccination rates compared to non-Latino Whites [14–16]. Recent online surveys regarding the COVID-19 vaccine have shown Latino intent to vaccinate is mixed, either demonstrating a similar rate or decreased likelihood to vaccinate compared to whites [17–21]. This is concerning because Latinos have higher rates of COVID-19 infection (1.7x) and mortality (2.8x) compared to whites [22]. Latinos aged 35–44 years are particularly impacted as they are eight times more likely to die of COVID-19 compared to their white counterparts, and six times more likely if they are 45–54 years-old [23]. This increased risk is potentially devastating since individuals in this age range are more likely to be supporting and caring for children and elderly family members. Factors including occupation (e.g., essential worker), lack of ability to physically distance, comorbidities (e.g., diabetes, hypertension, obesity, and lung disease), and decreased health care access likely contribute to this increased risk of infection and death [23].

Given their increased risk of infection and mortality, it is critically important to understand factors associated with Latino COVID-19 vaccine hesitancy. The goals of this mixed-methods study were to: (1) determine the intent to become vaccinated against COVID-19 in a primarily female, Latino, low-income population receiving aid from the Supplemental Nutrition Assistance Program (SNAP) in Southern California, (2) identify factors associated with vaccination intent, and (3) identify key concerns about potential COVID-19 vaccines. Information gathered in this study can help health providers and public health entities optimize their targeted health communication and increase vaccine receipt.

## Methods

### Sample and data collection

We implemented a cross-sectional survey in June and July 2020 to assess attitudes, behaviors, and perceived impact of COVID-19 on participants in the Southern California Nutrition Incentives Program, ¡Más Fresco! More Fresh. (Supplemental File 1) (ClinicalTrials.gov Identifier: NCT02976389). This program provides financial incentives to SNAP recipients to purchase more fresh fruits and vegetables at participating Northgate Gonzalez Markets, the largest Latino supermarket in Southern California [24]. A convenience sample of approximately 1556 out of 4500 program participants across San Diego, Orange, and Los Angeles Counties were invited to complete this survey; this was a targeted sample of participants who had already responded to at least one prior survey invitation. A total of 591 responded to this survey (response rate of 38%). Of these, 541 responded to the COVID-19 vaccine intent question, and 486 identified as Latino; this sample was the focus of the analysis. Based on participant preference, surveys were administered in English or Spanish over the phone by bilingual study staff or self-administered online via email or text message invitation. Qualtrics secure online survey platform was used to record responses for both administration methods. Skip patterns and non-responses led to missing data which are noted in Tables 1 and 2. Participants who did not respond to the question about intent to vaccinate against COVID-19 or had missing data from the independent variables were excluded from the multivariable analyses (Table 3). Respondents were given $50 store credit for fresh fruits and vegetables. This study was approved by the UC San Diego Human Research Protections Program as an amendment to the main intervention study and all participants completed informed consent. The STROBE guideline for cross-sectional studies was followed for reporting purposes [25].

**Table 1:** Latino participant characteristics stratified by COVID-19 vaccine intent (*n* = 486)

	ALL (***n*** = 486)		Definitely/ Likely Not (***n*** = 63)		Not sure/ Don’t Know (***n*** = 189)		Definitely/ Likely Yes (***n*** = 234)	
**SOCIODEMOGRAPHICS**	**Med.**	**IQR**	**Med.**	**IQR**	**Med.**	**IQR**	**Med.**	**IQR**
**Age (continuous, *n*** **= 486)**	40.0	13.0	36.0	12.0	39.0	12.0	40.0	13.0
	**No.**	**%**	**No.**	**%**	**No.**	**%**	**No.**	**%**
**Gender**								
Male	34	7%	3	5%	10	5%	21	9%
Female	451	93%	59	94%	179	95%	213	91%
Other/Missing	1	0%	1	2%	0	0%	0	0%
**Language Preference**								
English	128	26%	30	48%	49	26%	49	21%
Spanish	358	74%	33	52%	140	74%	185	79%
**Latino Origin**								
Latino, Mexican origin	425	87%	51	81%	165	87%	209	89%
Latino, not of Mexican origin	61	13%	12	19%	24	13%	25	11%
**Educational Attainment**								
High school graduate/GED or less	393	81%	45	71%	155	82%	193	82%
Some college or more	77	16%	14	22%	28	15%	35	15%
Missing	16	3%	4	6%	6	3%	6	3%
**Relationship Status**								
Married or living with partner	271	56%	24	38%	113	60%	134	57%
No partner (single, separated/divorced, widowed, other)	205	42%	38	60%	70	37%	97	41%
Missing	10	2%	1	2%	6	3%	3	1%
**Children in household**								
Any children aged 5–17 years	384	79%	48	76%	158	84%	178	76%
Any children aged 0–4 years	155	32%	27	43%	52	28%	76	32%
No children	76	16%	8	11%	26	14%	42	18%
Missing	2	0%	1	2%	1	1%	0	0%
**Food Security Categories, 6-item scale**								
High or marginal food security	151	31%	19	30%	62	33%	70	30%
Low or very low food security	311	64%	43	68%	117	62%	151	65%
Missing or did not answer at least 4 of 6 questions	24	5%	1	2%	10	5%	13	6%
**COVID-19 RISK FACTORS**								
**Are any adults living in the home an “essential worker?“**^a^								
Yes	159	33%	25	40%	66	35%	68	29%
No/Don’t know	327	67%	38	60%	123	65%	166	71%
**Any household health condition diagnoses**^b^								
No health conditions	159	33%	29	46%	50	26%	80	34%
Yes, but not a COVID-19 risk factor^c^	225	46%	28	44%	90	48%	107	46%
Yes, a COVID-19 risk factor^d^	71	15%	4	6%	33	17%	34	15%
Missing	31	6%	2	3%	16	8%	13	6%
**BMI Status**								
Normal BMI (< 25)	66	14%	9	14%	22	12%	35	15%
Overweight BMI (≥25 & < 30)	138	28%	21	33%	53	28%	64	27%
Obese BMI (≥30)	216	44%	28	44%	85	45%	103	44%
Missing	66	14%	5	8%	29	15%	32	14%
**Since COVID-19 began, how often do you see family members that you do not live with?**								
The same or more than before	82	17%	6	10%	39	21%	37	16%
Less than before or not at all	355	73%	49	78%	128	68%	178	76%
Missing	49	10%	8	13%	22	12%	19	8%
**Since COVID-19 began, how often do you see your friends?**								
The same or more than before	32	7%	4	6%	11	6%	17	7%
Less than before or not at all	412	85%	51	81%	162	86%	199	85%
Missing	42	9%	8	13%	16	8%	18	8%
**PREVIOUS INFLUENZA VACCINE USE**	159	33%	25	40%	66	35%	68	29%
**Have you ever received the flu vaccine in the past?**								
Yes	368	76%	33	52%	144	76%	191	82%
No	95	20%	28	44%	34	18%	33	14%
Missing	23	5%	2	3%	11	6%	10	4%
**Did you receive the flu vaccine this past flu season (2019–2020)?**								
Yes	257	54%	17	25%	102	60%	138	53%
No	203	40%	42	69%	76	36%	85	42%
Missing	26	6%	4	5%	11	4%	11	5%
**SOCIAL MEDIA USE AND COVID-19 ATTITUDES & BELIEFS**								
**Any social media use**								
Yes	411	85%	60	95%	162	86%	189	81%
No	54	11%	2	3%	17	9%	35	15%
Missing	21	4%	1	2%	10	5%	10	4%
**How often are you reading/talking about COVID-19?**								
Rarely/Never	310	28%	25	53%	128	28%	157	31%
Occasionally/Often/Most of the time	154	29%	34	24%	54	31%	66	29%
Missing	22	43%	4	23%	7	41%	11	39%
**COVID-19 Affect (Continuous, range: 1–5)**	**Mean**	**SD**	**Mean**	**SD**	**Mean**	**SD**	**Mean**	**SD**
**Mean Score**^e^**(*n*** **= 336)**	2.6	0.9	2.8	0.8	2.7	0.9	2.4	0.9

^a^Question also included the following text: “(e.g., healthcare, delivery worker, store worker, janitorial services, security, building maintenance)”

^b^Question text: “Has a health or educational professional ever told you that you or anyone who you live with have any of the following health conditions?”

^c^Response options not considered a COVID-19 risk factor: Allergies, arthritis, headaches, seizures/epilepsy, stomach/bowel issues, severe acne/skin issues, mental health, alcohol/drugs, intellectual disability, autism, learning disorder, other

^d^Response options that are considered COVID-19 risk factors: Asthma/lung issues, hypertension, high cholesterol, diabetes/high blood sugar, kidney issues

^e^Calculated only for participants answering at least 5 out of 10 questions. Cronbach’s alpha = 0.8226, item #3 (spreading slowly vs. fast) reverse-coded based on alpha item correlation. Data on the individual items in the COVID-19 Affect scale are available in Supplemental File 2, Supplemental Tables 1 & 2

**Table 2:** Unadjusted Odds Ratio for participants reporting they were unsure or unlikely to receive a COVID-19 vaccine (*n* = 336 to 486)^a^

SOCIODEMOGRAPHICS	Not Sure/ Don’t Know (vs. Definitely Yes/ Likely Yes)			Definitely Not/ Likely Not (vs. Definitely Yes/ Likely Yes)			Obs.
	OR	95% CI	***p***-Value	OR	95% CI	***p***-Value	
**Age (continuous)**	0.99	0.97–1.01	0.15	**0.96**	0.93–0.99	0.04	486
**Gender: Female** (vs. Male)	1.76	0.81–3.85	0.15	1.94	0.56–6.73	0.29	485
**Language Preference: English** (vs. Spanish)	1.32	0.84–2.08	0.22	**3.43**	1.91–6.17	0.001	486
**Latino not of Mexican origin** (vs. Latino of Mexican origin)	1.22	0.67–2.21	0.52	**1.96**	0.92–4.18	0.08	486
**Educational Attainment: Some college or higher** (vs. High school graduate/ GED or less)	0.99	0.58–1.71	0.99	1.71	0.85–3.45	0.13	470
**Marital Status: No partner- single, separated/divorced, widowed, other** (vs. married or living with partner)	0.86	0.57–1.27	0.44	**2.19**	1.23–3.88	0.008	476
**Any children aged 0–4** (vs. none)	0.79	0.52–1.21	0.28	1.60	0.90–2.84	0.111	484
**Any children aged 5–17** (vs. none)	**1.66**	1.01–2.71	0.04	1.08	0.55–2.10	0.82	484
**Low or Very Low Food Security, 6-item scale** (vs. High/marginal Food Security)	0.87	0.55–1.37	0.53	1.20	0.63–2.30	0.88	462
**COVID-19 RISK FACTORS**							
**Any adults living in the home are essential workers** (vs. none)	1.25	0.82–1.89	0.29	1.51	0.84–2.71	0.17	466
**Household members with lifetime health condition diagnoses** (vs. No household health diagnoses)							
Yes, but not a COVID-19 risk factor	1.35	0.86–2.11	0.20	0.72	0.40 1.31	0.28	455
Yes, a COVID-19 risk factor	1.55	0.86–2.82	0.15	**0.32**	0.11–0.99	0.049	
**BMI Status** (vs. Normal BMI (< 25))							
Overweight BMI (≥25 & < 30)	1.32	0.69–2.51	0.40	1.28	0.53–3.09	0.59	420
Obese BMI (≥30)	1.31	0.72–2.41	0.38	1.06	0.45–2.46	0.90	
**Since COVID-19 began, how often do you see family members that you do not live with? Less than before or not at all** (vs. the same or more than before)	0.68	0.41–1.13	0.14	1.70	0.68–4.26	0.26	437
**Since COVID-19 began, how often do you see your friends? Less than before or not at all** (vs. the same or more than before)	1.26	0.57–2.76	0.57	1.09	0.35–3.38	0.88	444
**PREVIOUS INFLUENZA VACCINE USE**							
**Never received the flu vaccine, lifetime** (vs. did receive)	1.37	0.81–2.31	0.25	**4.91**	2.63–9.18	0.001	463
**Did not receive the flu vaccine this past flu season (2019–2020)** (vs. did receive)	1.21	0.81–1.81	0.35	**4.01**	2.15–7.50	0.001	460
**SOCIAL MEDIA USE AND COVID-19 ATTITUDES & BELIEFS**							
**Any Social Media Platforms Used**^b^ (vs. I don’t use social media)	1.77	0.95–3.27	0.07	**5.56**	1.30–23.82	0.02	465
**Never/Rarely Reading/Talking about COVID-19** (vs. Occasionally/Most of the time/Often)	1.00	0.65–1.54	0.99	**3.23**	1.79–5.85	0.0001	464
**COVID-19 Affect Mean Score** (Continuous, range of 1–5)	**1.38**	1.06–1.81	0.02	**1.55**	1.11–2.17	0.009	336

^a^Robust 95% confidence intervals (CI). Each unadjusted OR was calculated for the existing sample that completed the question at hand. Odds ratios that are bolded indicate *p* < 0.10

^b^Social media platforms included Facebook, Twitter, Instagram, YouTube, Snapchat, WhatsApp, TikTok, Other

**Table 3:** Adjusted Odds Ratios (AOR) for participants reporting they were unsure or unlikely to receive a COVID-19 vaccine (*n* = 287)^a^

	Not Sure/ Don’t Know (vs. Definitely Yes/ Likely Yes) AOR (95% CI)	Definitely Not/ Likely Not (vs. Definitely Yes/ Likely Yes) AOR (95% CI)
**Age (continuous)**	0.98 (0.96–1.01)	0.98 (0.93–1.03)
**Language Preference: English** (vs. Spanish)	1.50 (0.83–2.71)	**3.05** (1.31–7.12)
**Latino not of Mexican origin** (vs. Latino of Mexican origin)	1.21 (0.54–2.75)	2.08 (0.71–6.13)
**No partner- single, separated/divorced, widowed, other** (vs. married or living with partner)	0.88 (0.51–1.53)	2.03 (0.88–4.71)
**Any children aged 5–17** (vs. none)	1.80 (0.83–3.89)	0.756 (0.26–2.17)
**Lifetime household health condition diagnoses** (vs. No household health condition diagnoses)		
Yes, but not a COVID-19 risk factor	1.48 (0.80–2.72)	1.10 (0.45–2.69)
Yes, a COVID-19 risk factor	**2.43** (1.05–5.64)	1.59 (0.41–6.10)
**Any Social Media Platforms Used (vs. I don’t use social media)**	1.15 (0.42–3.20)	1.25 (0.25–6.19)
**Never/rarely reading/talking about COVID-19** (vs. occasionally/often/most of the time)	0.89 (0.47–1.68)	**4.03** (1.72–9.43)
**Did not receive flu vaccine this past season (2019–2020)** (vs. did receive)	1.20 (0.70–2.04)	**3.14** (1.36–7.23)
**COVID-19 Affect Mean Score**	**1.38** (1.02–1.87)	1.40 (0.89–2.19)

^a^ Robust 95% confidence intervals (CI) in parentheses. Analysis was performed among those providing complete responses to all relevant questions

### Measures

#### Dependent variable

The primary dependent variable was vaccine intent. Participants were asked “if a COVID-19 vaccine is developed in the future, how likely are you to get it?” Response choices included a five-point Likert scale which was categorized into three groups: (0) “definitely yes/likely yes”, (1) “not sure/don’t know”, and (2) “definitely not/likely not.”

### Independent variables

Participants reported sociodemographic information including age, gender, marital status, education, children in the household, food security (6-item USDA module dichotomized into high vs. low and very low) [26], and race/ethnicity, which included identifying Latino of Mexican origin or non-Mexican origin.

Risk factors for COVID-19 infection included having an essential worker living in the household [27], a body mass index (BMI) of 25.0–29.9 (overweight) or ≥ 30 (obese), or a COVID-19 health-related risk factor. Participants were asked about multiple health conditions diagnosed in themselves or people in their household [27] and responses were grouped into three categories: no health conditions, one or more conditions not considered a COVID-19 risk factor, and one or more conditions considered a COVID-19 risk factor (e.g., asthma/respiratory disease, hypertension, high cholesterol, cardiovascular disease, diabetes, kidney disease) [28, 29]. Self-reported ability to physically distance was measured by contact with people outside their household and dichotomized (“less than before/not at all” or “same/more than before”). Prior influenza vaccine receipt was assessed with two questions: “ever had a flu vaccine” (i.e., lifetime vaccination) and “received the flu vaccine in the past season (2019-2020).”

To assess vaccine-related attitudes and influences, we asked if participants used any social media platforms (any vs. none) and how much they were reading or talking about COVID-19 (“rarely/never” or “occasionally/often/most of the time”) [27]. Finally, we used a 10-item measure to assess COVID-19 Affect, i.e., participants’ emotional reaction to COVID-19 related to the pandemic [30] (Supplemental File 1). This measure was adapted for COVID-19 from an existing validated measure [31], and was available for the research community on the WHO website. To our knowledge, the adapted COVID-19 version of this measure has yet to be validated as a single score variable. We calculated Cronbach’s alpha for the 10 items and found it to be acceptable (α = 0.82). Preliminary factor analyses yielded no discernable subscales. Weighted mean scores were calculated for participants who answered at least five out of 10 questions. The third item (spreading slowly vs. fast) was reverse-scored based on inter-item correlation. A higher score on the COVID-19 Affect scale was associated with a lower emotional response or concern with the COVID-19 pandemic. Data on the individual items in the COVID-19 Affect scale are available in Supplemental File 2: Supplemental Tables 1 & 2.

### Statistical analysis

Frequencies, means, and medians were used to present sociodemographic characteristics, vaccination intent, and COVID-19 related behaviors. Results were stratified by the dependent variable (COVID-19 vaccine intent). Since the dependent variable had three categorical values, multinomial logistic regressions were completed to identify factors significantly associated with participant vaccine intent. Although an ordinal logistic regression model was considered since there is some inherent order in the response categories, the data did not meet the assumption of proportional odds (i.e., the relationship between vaccine intent groups may not be the same). Moreover, the multinomial logistic regression approach allowed the reference group to be set to those who said they would “definitely yes/likely yes” get vaccinated; in this way, the differences between the reference group and those unsure of the vaccine could be compared with the differences between the reference group and those who were against receiving the vaccine. Univariate analyses were conducted to generate unadjusted odds ratios (OR) for each independent variable among those who would “definitely not/likely not” be vaccinated against COVID-19 compared to the reference group and for those who were “not sure/didn’t know” compared to the reference group.

All variables significant at *p* < 0.10 in the univariate logistic regression analyses were included in a multivariable multinomial logistic regression model to determine which factors were associated with unsure vaccine intent or negative vaccine intent compared to those with positive vaccine intent (i.e., adjusted odds ratios [AOR]). In specifying the final model, theoretical justification, impact on sample size (i.e., missing data), and correlation between predictor variables were considered. Lifetime influenza vaccination and influenza vaccination in 2019–2020 were highly correlated (*r* = 0.55), therefore only influenza vaccination in 2019–2020 was used in the multivariable model. Potential models were compared using Akaike information criterion. The final model’s goodness-of-fit was found to be appropriate in a Hosmer-Lemershow test adapted for multinomial logistic regression [32]. All analyses were conducted using Stata version 15.

### Qualitative analysis

Participants who indicated they definitely/likely would not or were unsure if they would get a potential COVID-19 vaccine were asked to briefly describe their thoughts in an open-ended question. Phone interview responses were recorded by study staff (*n* = 191). Qualitative thematic analysis methods were used to generate codes and identify themes and subthemes [33]. Two team members (SHV and YE, both bilingual) translated the Spanish language responses into English. They then conducted preliminary open coding on a subset (approximately 25%) of responses and developed a matrix framework for analysis in Microsoft Excel [34–36]. Two other team members (VPS and KER) reviewed codes, met with the full team to review discrepancies, and made final coding decisions collectively. More than one code could be assigned to a response. The data was recoded (YE) and SHV randomly checked 10% of the data to reconcile any remaining differences. The team then met to group codes into larger themes/subthemes, and frequencies were calculated.

## Results

### Sample characteristics

The median age of those responding to the vaccine intent question (*n* = 486) was 40.0 years (IQR, 13.0). The majority were female (93%), of Mexican origin (87%), and primarily Spanish speaking (74%) (Table 1). The majority (81%) also had a high school degree or less and had children living in the household (84%). Almost two-thirds of the sample reported low to very low food security levels. While few participants reported that they had a COVID-19 health risk factor (15%), 72% of the sample had overweight or obesity [29]. A third of the sample also reported that an “essential worker” lived in the home. While relatively few (20%) had never received a flu vaccine in the past, only 54% received a flu vaccine in the 2019–2020 influenza season. When asked about their intent to receive the COVID-19 vaccine, approximately half (48%) reported “definitely/likely yes”, 39% reported “not sure/don’t know,” and 13% reported “not likely/definitely not”.

### Factors associated with COVID-19 vaccine intent

In the unadjusted multinomial logistic regression models (Table 2), participants with a child between 5 and 17 years old in their household were more likely to report being unsure about getting a COVID-19 vaccine (Odds Ratio [OR] = 1.66, 95% Confidence Interval [CI] = 1.01–2.71). Those with a higher COVID-19 Affect score (indicating less concern with the virus) were also more likely to be unsure about getting the vaccine (OR = 1.38, CI = 1.06–1.81). Similarly, those with a higher COVID-19 Affect score were more likely to indicate that they would not get the vaccine (OR = 1.55, CI = 1.11–2.17). Those who primarily spoke English (OR = 3.43, CI = 1.91–6.17), were single/separated/divorced (OR = 2.19, CI = 1.23–3.88), did not receive the influenza vaccine last season (OR = 4.01, CI = 2.15–7.50), used social media platforms (OR = 5.56, CI = 1.30–23.82), or rarely talked about COVID-19 (OR = 3.23, CI = 1.79–5.85) were also more likely to report they would not get the vaccine. Those who were older (OR = 0.96, CI = 0.93–0.99) or had a household member with a COVID-19 health risk factor (OR = 0.32, CI = 0.11–0.99) were less likely to indicate that they would not get the vaccine. Significant findings at *p* < 0.10 included that Latinos not of Mexican origin were more likely to not want the vaccine compared to those of Mexican origin, and those who used social media platforms were more likely to be unsure of the vaccine (Table 2).

In the multivariable multinomial logistic regression model (Table 3), participants with a household member with a COVID-19 health risk factor were more likely to report being unsure about getting the vaccine (AOR = 2.43, CI = 1.05–5.64). A higher COVID-19 Affect score remained significant in this model, indicating that those who were less concerned about the virus were more likely to report they were unsure about getting the vaccine (AOR = 1.38, CI = 1.02–1.87). Participants who were primarily English speaking (AOR = 3.05, CI = 1.31–7.12), did not receive the influenza vaccine last season (AOR = 3.14, CI = 1.36–7.23), or reported not reading or talking about COVID-19 (AOR = 4.03, CI = 1.72–9.43) were more likely to report not intending to receive the vaccine (Table 3). This final model was repeated without the variable “received flu vaccine in the past season” and the remaining variables were still significant.

### Views of the COVID-19 vaccine

Participants who reported that they were unsure or unlikely to get a COVID-19 vaccine were asked to report their concerns about getting the vaccine. A little under half (*n* = 191) provided a brief written response or oral response via phone interview (Table 4). The most common theme (51% of respondents) focused on concerns about “side effects and ingredients.” Many were worried about an allergic reaction or that the vaccine might make them “sick,” cause them to “have a bad reaction and die,” or “get the [COVID-19] virus.” Some were concerned that the vaccine might contain toxic elements that would cause harm in the future. A few participants expressed more extreme beliefs that the vaccine included a “chip” that would track them and manipulate them.

**Table 4:** Concerns from participants who were unsure or unlikely to get a future COVID-19 vaccine (*n* = 191)

THEMES	SUB-THEMES	No.	%	EXAMPLE QUOTES FROM SPANISH & ENGLISH SPEAKERS *Original Spanish in italics, if applicable*
**Side effects/ ingredients (*n*** **= 97, 51%)**	Side effects/ allergic reactions	40	21%	**“I do not know the side effects and neither do I know what it is made of.”***No se los efectos secundarios y tampoco se de que está echa* **“I feel a bit unsure of the reactions my body may have after receiving it.”***Me siento un poco insegura de las reacciones que pueda tener mi cuerpo después de recibirla.* **“That I have a bad reaction and die.”***De que tenga una reacción mala y me muera.* **“If it’ll make me sick or have illness as a side effect.”** **“That it will get me allergic.”**
	Will cause illness/ COVID-19	31	16%	**“I would be afraid of getting the vaccine and even more afraid if getting the vaccine means I’ll get the virus”***Me daría miedo ponérmela porque peor si es para que me dé.* **“I think the vaccine will bring more risks for one to get sick.”***Pienso que la vacuna traerá más riesgos a que uno se enferme* **“That it infects me with COVID-19, I don’t trust anyone.”***Que me contagie COVID-19, no confío en nadie*
	Concern about ingredients	13	7%	**“Because we do not know the harmful chemicals that they are going to contain and in the future may have secondary reactions …**.” *Porque no sabemos los químicos perjudiciales que vayan a contener y en el futuro puedan tener reacciones secundarias, y por otras razones* **“I don’t know the contents of the vaccine and if they are toxic.”** **“You don’t know what is in the vaccine.”** **“Not really sure since they have to add the virus to create and anti- virus, I’m not just gonna give it to my family that means it contains the virus so no.”**
	Will cause extreme harm/ death/ has a chip	12	6%	**“That they will implant a chip in her.”***Que se implante un chip en ella.* **“That supposedly it comes with a chip to manipulate people.”***El que según es con chip para manejar ala gente.* **“That the vaccine is to make a person die, not to cure them.”***Que la vacuna es para que se muera uno, no para curar*. **“Being in pharmaceuticals, I don’t think that they can come up with a complete, safe vaccine in 18 months to prevent this. It’ll take a lot longer. The way I’m looking at the research, I think they’re hurrying because they want to test it on people. If it doesn’t work, I’m afraid it will kill people and I don’t want to put myself or my children at risk for that.”**
	Other vaccines/ flu shot made them sick or didn’t work	7	4%	“**Once, I got very sick after getting a vaccine. So that’s why I avoid all vaccines. Now, I don’t get vaccinations and I never get sick.”***Una vez, me enferme mucho después de recibir una vacuna. Entonces, por eso evito todas las vacunas. Ahora, no recibo las vacunas y nunca me enfermo* “**In the past, I have had vaccines and I have had very strong reactions (from the flu, hepatitis vaccines). Well, a doctor told me it was due to the vaccines. So, I’m not sure about the vaccines.”***En tiempo pasada, me puesto vacunas y me han reacciones muy fuertes (la de flu, hepatitis). Pues, una doctora me dijo que fue debido a las vacunas. Entonces, no estoy seguro de las vacunas* **“I got sick from the flu vaccine.”** **“I am afraid that the vaccine might make me sick. I have experiences with other vaccines that have made me sick.”**
**Distrust/ Uncertainty (*n*** **= 91, 48%)**	Fearful/ distrusting, general	38	20%	**“I don’t know if it will be safe.”***No se si vaya a ser seguro* **“That it is really for that [COVID-19] and not for another purpose.”***De que sea realmente para eso y no para otro fin.* **“A little fear and distrust because everyone is rushing to find a vaccine.”***Un poco de temor y desconfianza porque todos se estan apurando para encontar una vacuna.* **“There is controversy over the vaccine and I have heard a lot of negative things about that vaccine and I would be afraid if I or a loved one got sick because of the vaccine.”**
	More testing needed/wait to see what happens	34	18%	“**Because people tell me that it is not good to receive it because the doctors are pressuring themselves to find the vaccine.”***Porque la gente me dice que no es buena recibirla porque los doctores se están apresurando para buscar la vacuna* **That it will be something new. I’m not sure I want a vaccine that is not well developed.”***Que es algo nuevo. No estoy segura de querer una vacuna que aun no esté bien desarrollada* **“It is very quick to know if it will work correctly.”***Es muy rápido para saber si funcionara de manera correcta* **“I wouldn’t get it because I don’t think there is enough research or evidence and I wouldn’t feel safe afterwards.”** **“It’s a new medication that hasn’t been tested long enough.”** **“It has been rushed and I don’t know if I will want it until it has been out for a short while.”** **“I would like to wait until year 2 to see if there are any serious side effects.”**
	Unsure/ need more info	30	16%	**“She would like to know more information about the vaccine because she thinks this virus is stronger than anything out there.”** **“I need to be well informed about the vaccine and how it would affect me and my family.”** **“Not sure about getting the COVID-19 because it would be new and she would not know anything about it.”**
**Effectiveness (*n*** **= 30, 16%)**	Whether the vaccine will work	30	16%	**“That it will be worse and it doesn’t work.”***Que sea peor y no funcione.* **“That it will not be the correct one.”***Que no sea la correcta.* “**That they don’t come out with the correct one and still get infected.”** **“See if it works or not!!!”** **“I am afraid it might not work”**
**General dislike of vaccines (*n*** **= 22, 12%)**	General dislike of vaccines	15	8%	**“Allergic reaction or unnecessary injections. The flu vaccine doesn’t improve anything. I get sick more during the year when I’ve had it. This runs in my fam. I’ve seen people who get sick often if they don’t get it. Not everyone needs it.”** **“There are so many vaccines out there that is not need and don’t do nothing. I believe if we strengthen our immune system our bodies is capable to fight disease.”** **“I don’t like the vaccines.”** **“Has there been testing on it and will it prevent it from coming back because the flu vaccine I still got the flu. It’s alarmist like taking Tylenol without a headache.”** **“I am not 100% confident in vaccines being healthy to take. Scared of side effects. I am 56 and only had the flu shot like 3 times.”**
	Already healthy/ will take alternative pre-cautions	11	6%	**“None but I would not allow them to give me the vaccine because I am in good health”***Ninguna pero no permitiría que me pusieran la vacuna porque estoy bien de salud.* **“Wearing a mask, taking care to go out only when necessary”***Usar tapabocas cuidarse salir no más cuando es necesario.* **“Will not get vaccine because she believes her immune system is strong”** **“I rather take precautions such as washing my hands and not touch my face than take the vaccine.”** **“There is so many vaccines out there that are not needed and don’t do nothing. I believe if we strengthen our immune system our bodies are capable to fight disease.”**

Asked of those who said “not sure,” “not likely,” or “definitely not” to the question about their likelihood of getting a COVID-19 vaccine. Quotes written in third person are based on notes taken by study staff during phone interviews

Close to half the respondents (48%) had a general distrust of the vaccine and the vaccine-making process. Many participants were concerned about the speed of vaccine development and testing, and expressed a desire to wait and see what happens to others who get the vaccine. Overall, there was a general desire for more information about COVID-19 vaccines before making a decision.

A smaller proportion of participants (16%) described concerns about “effectiveness” and whether the vaccine would work at all or as intended. Several reported getting “the flu” despite getting the influenza vaccine, so they did not want to get the COVID-19 vaccine. Others reported they were healthy with a strong immune system or preferred taking other precautions (mask wearing, hand washing, not going out unless necessary), so they would not need this vaccine. Finally, a small group of participants (12%) expressed a “general dislike/distrust of vaccines,” and therefore would not trust this vaccine.

## Discussion

Among this Latino, primarily female, low-income population in Southern California, only half (48%) reported an intent to vaccinate against COVID-19. This rate is lower than that reported in other studies collecting data at a similar time (April–June 2020: 58–79% [17–21]), and may reflect the uniqueness of this sample. Females typically report lower COVID-19 vaccine intent compared to males [17, 21], and in past studies Latinos have had lower overall vaccination rates compared to non-Latino Whites [14–16]. Latino women/mothers are often the decision-maker regarding vaccinations for their family [37]. Addressing their concerns and understanding their views will be important in the development of relevant and effective public health messages.

One factor associated with decreased intent to receive a COVID-19 vaccine was English language preference. This finding adds to previous literature that demonstrates variation in vaccination rates based on language preference and country of origin, and raises the question of whether linguistic isolation leads to varied exposure to Western vaccine views [16, 37]. Latina mothers traditionally follow the recommendations of physicians [38]. However, as they spend more time in the US, they may become more acculturated and influenced by predominantly English language informational sources (e.g., social media platforms and online websites), thereby increasing their exposure to anti-vaccine sentiments [37]. Development of targeted educational interventions for Latinos (in English and Spanish) that address anti-vaccine views and highlight the safety and efficacy of vaccines are needed when implementing culturally sensitive health communication strategies [39].

Similar to other studies, participants who did not get the influenza vaccine in the 2019–2020 season were less likely to want the COVID-19 vaccine [17, 20]. Therefore, healthcare workers may want to consider using prior influenza vaccine refusal as an indicator of COVID-19 vaccine hesitancy and initiate early discussions about vaccine concerns with these patients. In the qualitative results, many commented on the lack of effectiveness of the influenza vaccine, stating that they “still got the flu even if they got the vaccine.” Since many in the community at that time had successfully protected themselves from getting COVID-19 by practicing physical distancing or wearing a mask, they could view these behaviors as sufficient. Public health efforts may need to highlight the differences between influenza and COVID-19 vaccine efficacy to encourage greater uptake of the COVID-19 vaccine. Even though concern or fear about COVID-19 was not significantly associated with intent to vaccinate in the final model, emphasizing the increased morbidity and mortality of COVID-19 for Latinos may help increase risk perception [40], the urgency of prevention, and promote vaccine acceptance.

We also found that those who were not reading or talking about COVID-19 were less likely to get vaccinated. However, it should be noted that 85% of this population use a social media platform, which may be another means to disseminate accurate COVID vaccine information. Research that identifies which information sources are trusted among various sub-groups of this low-income, Latino population will be important to establishing effective communication avenues. Furthermore, research to determine what type of messaging should be used to motivate Latinos to get a COVID-19 vaccine should be conducted to increase vaccination receipt.

A little over a third of the participants reported being unsure about getting the vaccine. This may be a particularly important group to target. The primary factor associated with being unsure was having a household member with a COVID-19 risk factor. This finding differs from an international survey study by Solis Arce, et al. who found that after personal protection, participants reported that a reason to vaccinate against COVID-19 is to protect their family [41]. Our qualitative results suggest that many participants were concerned about the expedited timeline of vaccine development and vaccine side effects (e.g., “have a bad reaction and die”, Table 4), which is similar to other studies performed in the US, as well as in low- and middle-income countries [41, 42]. Thus, participants may not want to put their family members at risk, especially if they are thought to be more vulnerable to illness, which highlights the family-centered thinking (familismo) that is common in Latino culture [43]. More culturally sensitive education may therefore be needed around how vaccines work (i.e., that getting the vaccine does not mean that someone is now infectious and can spread the virus), that having a COVID-19 risk factor does not mean someone will react poorly or become sicker when they get the vaccine, and that protection incurred after vaccination will likely outweigh any side effects one may experience. It has been reported that lack of vaccine-related knowledge or lower socioeconomic status are linked to decreased likelihood to vaccinate against COVID-19 [17, 38, 44, 45]. This further highlights the importance of improving how healthcare workers and public health organizations deliver information that addresses the Latino community’s concerns. Since long term effects of the vaccines are still unknown, educational platforms with intermittent, updated, and transparent safety data, that specifically focus on post-vaccination side effects, may be key to establishing trust in the public and reaching herd immunity [46]. It will be important to ensure that educational efforts are culturally sensitive and targeted for a variety of populations [14, 16, 47, 48].

### Limitations

While this study provides unique insight into the views of low-income, primarily female, Latino SNAP participants, there were some limitations. Missing data may have affected our ability to conduct multivariable regressions since participants were allowed to leave questions unanswered or did not complete questions because of skip patterns in the survey. Furthermore, these results may not translate to other Latino sub-groups in other areas of the country. Previous studies have found differences in sub-group analysis among Latinos based on birthplace and education [37]. So while it is important to explore reasons for vaccine hesitancy in the Latino population overall, further research examining sub-group differences is warranted to ensure successful public health campaigns that increase vaccine receipt for a diverse sector of the population.

## Conclusion

In this group of low-income, primarily female, Latino SNAP participants in Southern California, approximately half reported intent to vaccinate against COVID-19. High levels of distrust about vaccine content, side effects, and the expedited timeline to production persist among this population. Since the time of this survey, more information has been made available regarding the safety and efficacy of several vaccines, and vaccine intent may have increased. As more information about vaccine safety and its longer-term effects are made available, it will be important to disseminate this information to at-risk communities and increase their immunity to this deadly disease.

## Supplementary Information

**Additional file 1.** Supplemental Survey. Más Fresco! More Fresh Participant COVID-19 Survey. Survey items answered by participants.

**Additional file 2: Supplemental Table 1.** COVID-19 Affect Scale Individual Items Stratified by COVID-19 Vaccine Intent. **Supplemental Table 2.** Unadjusted Odds Ratio (OR) for participants reporting they were unsure or unlikely to receive a COVID-19 vaccine, COVID-19 Affect Scale Individual Items.

## Data Availability

De-identified datasets used in this analysis may be made available for scientific purposes upon reasonable request to the corresponding author.

## Abbreviations

COVID-19: Coronavirus Disease 2019
SNAP: Supplemental Nutrition Assistance Program
BMI: Body Mass Index
