# Supplementary material for: Factors associated with COVID-19 vaccine intent among Latino SNAP participants in Southern California
Source: BMC Public Health. 2022 Apr 5;22:653. doi: 10.1186/s12889-022-13027-w (PMC8981200; doi:10.1186/s12889-022-13027-w)
Supplement: Supplementary file 1 — Additional file 1. Supplemental Survey. Más Fresco! More Fresh Participant COVID-19 Survey. Survey items answered by participants. [file 12889_2022_13027_MOESM1_ESM.pdf]

Supplemental File 1:

¡Más Fresco! More Fresh Participant COVID-19 Survey[1]

To start, we'd like to learn a little bit about you and your family.

DEMOGRAPHICS

1. How old are you?
*Write your age in years:*_________

2. Are you...

- ☐ Female
- ☐ Male
- ☐ Other- please specify: ____
- ☐ Don't know
- ☐ Prefer not to answer

3. What is your race or ethnicity?
Select all that apply.

- ☐ Hispanic, Latino, or Spanish
- ☐ White
- ☐ Black/African American
- ☐ American Indian or Alaska Native
- ☐ Asian
- ☐ Native Hawaiian or Other Pacific Islander
- ☐ Middle Eastern or North African
- ☐ Some other race/ethnicity- write in below: ____
- ☐ Don't know
- ☐ Prefer not to answer

---

[1] Spanish translations of these items are available from the authors upon request.

4. What is your Hispanic, Latino, or Spanish origin?
Select all that apply.

- □ Mexican, Mexican American, Chicano
- □ Salvadoran
- □ Guatemalan
- □ Honduran
- □ Another Hispanic, Latino, or Spanish origin- write in origin, for example, Puerto Rican, Cuban, Argentinian, Colombian, Dominican, Nicaraguan, Costa Rican, Spaniard, and so on. Please specify below:
  ____
- □ Don't know
- □ Prefer not to answer

5. Are you currently…

- □ Single, never married
- □ Married
- □ Not married but living with a partner
- □ Widowed
- □ Divorced
- □ Separated
- □ Other- please specify: ____
- □ Don't know
- □ Prefer not to answer

6. What is the highest grade or level of school you have completed or the highest degree you have received?

- □ 8th grade or less
- □ Some high school, no diploma
- □ High school graduate/GED
- □ Some college, no degree
- □ Associates degree (2 year college)
- □ Bachelors degree (4 year degree, examples: BA, AB, BS, BBA)
- □ Masters Degree (example: MA, MS, MENG, MED, MBA)

- □ Doctoral Degree (example: MD, DDS, DVM, JD, PhD, EDD)
- □ Don't know
- □ Prefer not to answer

7. How tall are you? If you do not know, please provide your best estimate.
Enter height in feet/inches or in meters/centimeters

Feet ____

Inches ____

Meters ____

Centimeters ____

8. How much do you weigh? If you do not know, please provide your best estimate.
Enter your weight in pounds or kilograms

Pounds ____

Kilograms ____

9. Including yourself, how many of the people in your household are in each of the following age groups...
Fill in the number of people in each age group in your household.

Adults age 18 to 64? ____

Adults age 65 or older? ____

Children between the ages of 5 and 17 years? ____

Children under 5 years of age? _______________________________________

10. What social media do you use (if any):
Select all that apply

- □ Facebook
- □ Twitter
- □ Instagram
- □ YouTube
- □ Snapchat
- □ WhatsApp
- □ TikTok

- □ Other: ____
- □ I don’t use social media
- □ Don't know/Prefer not to answer

11. Here are several statements that people have made about their food situation. Please indicate if these statements are often true, sometimes true, or never true about your household since COVID-19 stay-at-home orders began on March 13, 2020

Since the COVID-19 crisis began, the food that (I/we) bought just didn’t last, and (I/we) didn’t have money to get more.

- □ Often true
- □ Sometimes true
- □ Never true
- □ Don't know
- □ Refuse to answer

12. Since the COVID-19 crisis began, (I/we) worried whether our food would run out before (I/we) got money to buy more.

- □ Often true
- □ Sometimes true
- □ Never true
- □ Don't know
- □ Refuse to answer

13. Since the COVID-19 crisis began, (I/we) couldn’t afford to eat balanced meals.

- □ Often true
- □ Sometimes true
- □ Never true
- □ Don't know
- □ Refuse to answer

14. Since the COVID-19 crisis began, did you or other adults in your household ever cut the size of your meals or skip meals because there wasn't enough money for food?

- □ Yes
- □ No
- □ Don't know

- □ Refuse to answer

15. How often did this happen—almost every month, some months but not every month, or in only 1 or 2 months?

- □ Almost every month
- □ Some months but not every month
- □ Only 1 or 2 months
- □ Don't know
- □ Refuse to answer

16. Since the COVID-19 crisis began, did you ever eat less than you felt you should because there wasn't enough money for food?

- □ Yes
- □ No
- □ Don't know
- □ Refuse to answer

17. Since the COVID-19 crisis began, were you ever hungry but didn't eat because there wasn't enough money for food?

- □ Yes
- □ No
- □ Don't know
- □ Refuse to answer

18. Are any adults living in the home an ESSENTIAL WORKER (e.g., healthcare, delivery worker, store worker, janitorial services, security, building maintenance)?

- □ Yes
- □ No
- □ Don't know
- □ Refuse to answer

19. Has a health or educational professional ever told you that you or anyone who you live with have any of the following health conditions?

Select all that apply

- □ Seasonal allergies

- ☐ Asthma or other lung problems
- ☐ Heart problems
- ☐ High blood pressure
- ☐ High cholesterol
- ☐ Kidney problems
- ☐ Immune disorder
- ☐ Diabetes or high blood sugar
- ☐ Cancer
- ☐ Arthritis
- ☐ Frequent or very bad headaches
- ☐ Epilepsy or seizures
- ☐ Serious stomach or bowel problems
- ☐ Serious acne or skin problems
- ☐ Emotional or mental health problems such as Depression or Anxiety
- ☐ Problems with alcohol or drugs
- ☐ Intellectual disability
- ☐ Autism Spectrum Disorder
- ☐ Learning Disorder
- ☐ Other condition (please specify) ____
- ☐ None of these
- ☐ Don't know
- ☐ Refuse to answer

20. These questions are about vaccines.
Have you ever received the flu vaccine in the past?

- ☐ Yes
- ☐ No
- ☐ Don't know
- ☐ Refuse to answer

21. Did you receive the flu vaccine this past flu season (2019-2020)?

- ☐ Yes
- ☐ No

- ☐ Don't know
- ☐ Refuse to answer

22. If they develop a COVID-19 vaccine in the future, how likely are you to get it?

- ☐ Definitely yes
- ☐ Likely yes
- ☐ Not sure
- ☐ Not likely
- ☐ Definitely not
- ☐ Don't know
- ☐ Refuse to answer

23. What are your concerns about getting a COVID-19 vaccine? Please write them below.

____

24. Compared to before the COVID-19 crisis began, how often are you seeing the following people in your life in person?

	Not at all	Less than before	About the same	More than before	Not applicable	Don't know	Refuse to answer
**Your family/people you live with**	☐	☐	☐	☐	☐	☐	☐
**Family members who you don't live with**	☐	☐	☐	☐	☐	☐	☐
**Friends**	☐	☐	☐	☐	☐	☐	☐
**Co-workers**	☐	☐	☐	☐	☐	☐	☐

25. How much are you reading or talking about Coronavirus/COVID-19?

- □ Never
- □ Rarely
- □ Occasionally
- □ Often
- □ Most of the time
- □ Don't know
- □ Refuse to answer

26. Please choose one option per row below. COVID-19 to me feels ...

	1	2	3	4	5	
Close to me	□	□	□	□	□	Far away from me
New	□	□	□	□	□	Old
Spreading slowly	□	□	□	□	□	Spreading fast
Something I think about all the time	□	□	□	□	□	Something I almost never think about
Fear-inducing	□	□	□	□	□	Not fear-inducing
Media-hyped	□	□	□	□	□	Not media-hyped
Worrying	□	□	□	□	□	Not worrying
Something that makes me feel helpless	□	□	□	□	□	Something that I am able to combat with my own action
Stressful	□	□	□	□	□	Not stressful
Something that is making me depressed	□	□	□	□	□	Something that does not affect my mood
