# Supplementary material for: Factors associated with COVID-19 vaccine intent among Latino SNAP participants in Southern California
Source: BMC Public Health. 2022 Apr 5;22:653. doi: 10.1186/s12889-022-13027-w (PMC8981200; doi:10.1186/s12889-022-13027-w)
Supplement: Supplementary file 2 — Additional file 2: Supplemental Table 1. COVID-19 Affect Scale Individual Items Stratified by COVID-19 Vaccine Intent. Supplemental Table 2. Unadjusted Odds Ratio (OR) for participants reporting they were unsure or unlikely to receive a COVID-19 vaccine, COVID-19 Affect Scale Individual Items. [file 12889_2022_13027_MOESM2_ESM.pdf]

SUPPLEMENTAL FILE 2

Supplemental Table 1: COVID-19 Affect Scale Individual Items Stratified by COVID-19 Vaccine Intent (n=486)

	**ALL (n=486)**		**Definitely/ Likely Not (n=63)**		**Not sure/ Don't Know (n=189)**		**Definitely/ Likely Yes (n=234)**	
**COVID-19 Affect (Continuous, range: 1-5)**	**Mean**	**SD**	**Mean**	**SD**	**Mean**	**SD**	**Mean**	**SD**
Close to me *1 2 3 4 5* Far away from me (n=326)	3.3	1.5	3.6	1.5	3.6	1.4	3.1	1.5
New *1 2 3 4 5* Old (n=337)	2.2	1.4	3.0	1.6	2.2	1.5	2.1	1.3
Spreading slowly *1 2 3 4 5* Spreading fast (n=326, reversed for scoring)	3.7	1.4	3.9	1.3	3.7	1.5	3.7	1.4
Something I think about all the time *1 2 3 4 5* Something I almost never think about (n=323)	2.9	1.4	3.2	1.4	3.0	1.3	2.7	1.4
Fear-inducing *1 2 3 4 5* Not fear-inducing (n=339)	2.4	1.4	2.5	1.3	2.6	1.5	2.2	1.4
Media hyped *1 2 3 4 5* Not media hyped (n=332)	2.6	1.4	2.5	1.4	2.7	1.5	2.4	1.4
Worrying *1 2 3 4 5* Not worrying (n=350)	2.1	1.4	2.4	1.3	2.3	1.5	1.9	1.3
Something that makes me feel helpless *1 2 3 4 5* Something I am able to combat with my own action (n=329)	2.7	1.5	2.8	1.3	2.9	1.5	2.5	1.4
Stressful *1 2 3 4 5* Not stressful (n=340)	2.3	1.4	2.6	1.4	2.4	1.4	2.1	1.3
Something that is making me depressed *1 2 3 4 5* Something that does not affect my mood (n=333)	2.8	1.4	2.8	1.4	2.9	1.5	2.8	1.5

Note: Reference variable is underlined.

Supplemental Table 2: Unadjusted Odds Ratio (OR) for participants reporting they were unsure or unlikely to receive a COVID-19 vaccine, COVID-19 Affect Scale Individual Items (n=323 to 350)[a]

	**Not Sure/ Don't Know** (vs. Definitely Yes/ Likely Yes)			**Definitely Not/ Likely Not** (vs. Definitely Yes/ Likely Yes)			**Obs.**
	**OR**	**95% CI**	**p-Value**	**OR**	**95% CI**	**p-Value**	
Close to me *1 2 3 4 5* Far away from me	**1.23**	1.05 - 1.44	0.01	**1.23**	0.97 - 1.57	0.08	326
New *1 2 3 4 5* Old	1.08	0.91 - 1.27	0.38	**1.52**	1.21 - 1.90	0.00	337
Spreading slowly *1 2 3 4 5* Spreading fast	0.97	0.83 - 1.15	0.74	1.11	0.87 - 1.40	0.40	326
Something I think about all the time *1 2 3 4 5* Something I almost never think about	1.13	0.95 - 1.33	0.17	**1.30**†	1.00 - 1.68	0.05	323
Fear-inducing *1 2 3 4 5* Not fear-inducing	**1.17**	0.99 - 1.38	0.06	1.12	0.90 - 1.40	0.30	339
Media hyped *1 2 3 4 5* Not media hyped	**1.16**	0.99 - 1.37	0.08	1.03	0.82 - 1.29	0.79	332
Worrying *1 2 3 4 5* Not worrying	**1.24**	1.04 - 1.47	0.02	**1.33**	1.07 - 1.66	0.01	350
Something that makes me feel helpless *1 2 3 4 5* Something I am able to combat with my own action	**1.21**	1.03 - 1.43	0.02	1.19	0.96 - 1.46	0.11	329
Stressful *1 2 3 4 5* Not stressful	1.14	0.96 - 1.36	0.12	**1.27**	1.01 - 1.59	0.04	340
Something that is making me depressed *1 2 3 4 5* Something that does not affect my mood	1.01	0.86 - 1.19	0.86	1.01	0.80 - 1.26	0.97	333

Note: Reference variable is underlined.

[a] Robust 95% confidence intervals (CI). Each unadjusted OR was calculated for the existing sample that completed the question at hand. Odds ratios that are **bolded** indicate p<0.10.
